# SMIFH2 inhibition of platelets demonstrates a critical role for formin proteins in platelet cytoskeletal dynamics

**DOI:** 10.1111/jth.14735

**Published:** 2020-02-17

**Authors:** Hannah L. H. Green, Malou Zuidscherwoude, Fawaz Alenazy, Christopher W. Smith, Markus Bender, Steven G. Thomas

**Affiliations:** ^1^ Institute of Cardiovascular Sciences University of Birmingham Birmingham UK; ^2^ Centre of Membrane Proteins and Receptors (COMPARE) University of Birmingham and University of Nottingham Midlands UK; ^3^ Institute of Experimental Biomedicine – Chair I University Hospital and Rudolf Virchow Center Würzburg Germany; ^4^ Present address: School of Cardiovascular Medicine & Sciences BHF Centre of Research Excellence King's College London London UK

**Keywords:** actin, blood platelets, formin, SMIFH2, tubulin

## Abstract

**Background:**

Reorganization of the actin cytoskeleton is required for proper functioning of platelets following activation in response to vascular damage. Formins are a family of proteins that regulate actin polymerization and cytoskeletal organization via a number of domains including the FH2 domain. However, the role of formins in platelet spreading has not been studied in detail.

**Objectives:**

Several formin proteins are expressed in platelets so we used an inhibitor of FH2 domains (SMIFH2) to uncover the role of these proteins in platelet spreading and in maintenance of resting platelet shape.

**Methods:**

Washed human and mouse platelets were treated with various concentrations of SMIFH2 and the effects on platelet spreading, platelet size, platelet cytoskeletal dynamics, and organization were analyzed using fluorescence and electron microscopy.

**Results:**

Pretreatment with SMIFH2 completely blocks platelet spreading in both mouse and human platelets through effects on the organization and dynamics of actin and microtubules. However, platelet aggregation and secretion are unaffected. SMIFH2 also caused a decrease in resting platelet size and disrupted the balance of tubulin post‐translational modification.

**Conclusions:**

These data therefore demonstrated an important role for formin‐mediated actin polymerization in platelet spreading and highlighted the importance of formins in cross‐talk between the actin and tubulin cytoskeletons.

**Essentials**

- Actin dynamics are critical for proper platelet function.
- We established the contribution of formin proteins in platelets using an FH2 domain inhibitor.
- Formins are required for spreading and organization of the actin and microtubule cytoskeletons.
- Formin function is needed for maintenance of resting platelet size and tubulin ring acetylation.

## 1 INTRODUCTION

Platelets require a dynamic actin and tubulin cytoskeleton for proper maintenance of their resting size and shape, and for their response to vascular damage. Formins are a family of 15 actin‐nucleating factors that promote the assembly of linear actin filaments downstream of the Rho family of small GTPases.^1^ Formin proteins contain a number of domains including FH1 and FH2^1^ and are known to function as homodimers.^2^ Profilin‐bound actin monomers associate with the FH1 domain of the formin dimer and are added to the growing actin filament by the action of FH2 domains, which act to both elongate the filament and protect it from capping proteins.^3^, ^4^, ^5^ In addition to their role in nucleating actin filaments, formins have been shown to play an important role in microtubule organization and dynamics, including alignment of actin and microtubules, microtubule stabilization, and microtubule bundling^6^, ^7^ indicating a role for formin proteins in coordination of actin and microtubule cytoskeletons in cells. Furthermore, changes in formin protein activity have been associated with human disease including hearing loss, cancer invasion, and neuropathy.^8^, ^9^, ^10^, ^11^, ^12^

We have previously reviewed expression databases for the 15 formin proteins in developing megakaryocytes and platelets at both the DNA and protein level.^7^ We reported that only four of the 15 formins were expressed in human or mouse platelets; these being DAAM1, mDia1, FHOD1, and INF2 (see table 1 of Zuidscherwoude et al^7^ for relative expression levels). The presence of DAAM1, mDia1, and FHOD1 has been confirmed in platelets by western blotting.^13^ Studies on the mDia1 knockout mouse indicated no major phenotype in terms of platelet activation, aggregation, or spreading, possibly due to functional redundancy between the expressed formin members;^13^ however, blocking mDia1 function in platelets using anti‐mDia1 antibodies was shown to reduce platelet spreading.^14^ mDia1 has been shown to play a role in megakaryocyte development and proplatelet formation (PPF) as knockdown of mDia1 using shRNA resulted in increased PPF.^15^ Further studies on gain of function mutations in mDia1 have shown that constitutively active mDia1 leads to reduced PPF, and patients expressing gain of function mutations display (macro)thrombocytopenia.^15^, ^16^, ^17^ Despite these studies, the contribution of formin proteins to platelet actin and microtubule organization remains unclear.

SMIFH2 was identified in 2008 by screening small molecules for their ability to inhibit actin assembly by mDia1 and mDia2.^18^ This activity was attributed to the FH2 domain, as the ability to inhibit actin polymerization persisted when profilin and the FH1 domain were absent, and therefore SMIFH2 is a useful tool to block the FH2 domains of all expressed formins. In mammalian fibroblasts, SMIFH2 inhibited formin‐dependent migration and reduced membrane integrity, but had little effect on Arp2/3 complex‐dependent structures such as lamellipodia.^18^ However, the effects of FH2 domain inhibition on the platelet cytoskeleton are not defined.

In the current study, human and mouse platelets were visualized with fluorescence and electron microscopy to observe changes to morphology and actin and tubulin dynamics in response to SMIFH2 treatment. Assays were performed with resting platelets, platelets undergoing spreading in response to activation by fibrinogen, and in platelet aggregate formation under flow conditions. Furthermore, the stability of microtubules following SMIFH2 treatment was investigated by fluorescence microscopy and western blotting to detect post‐translationally modified populations of *α*‐tubulin in resting platelets, indicative of stable and dynamic forms of the protein.

## 2 METHODS

Full detailed methods are provided in the Supporting Information.

## 3 RESULTS

### 3.1 Effect of global formin inhibition on platelet function

We had previously hypothesized that the lack of a phenotype in the mDia1 knockout (KO) mice was due to a redundancy with other formin proteins expressed in platelets.^13^ We tested this by treating both mouse and human platelets with a global formin inhibitor (SMIFH2) that blocks the FH2 domain of formins^18^ to establish if formin activity was required for proper platelet function.

### 3.2 Mouse platelets

**Figure 1:**
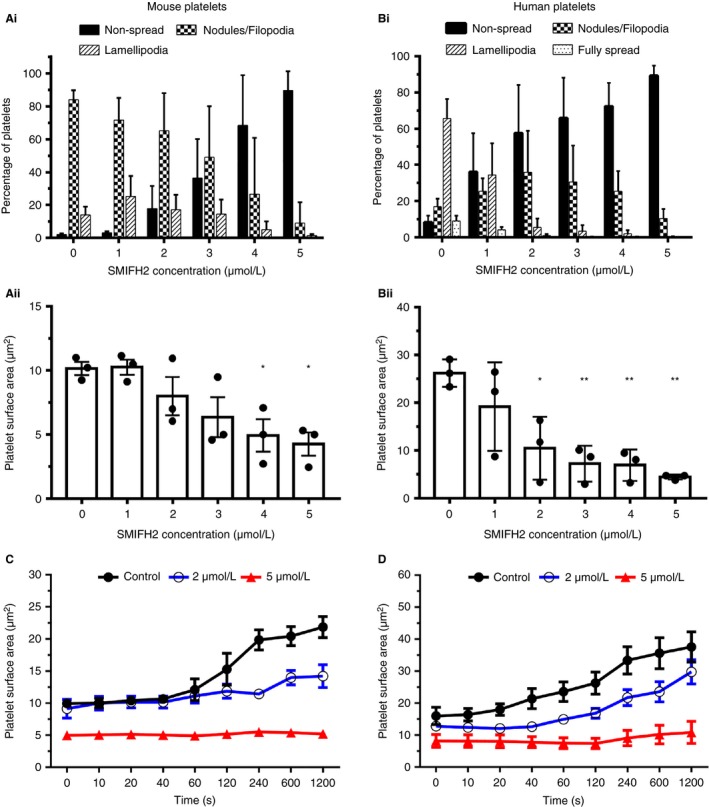
Inhibition of FH2 domains blocks platelet spreading. Ai & Bi, Analysis of spread platelet morphology (mean ± standard deviation) and (Aii & Bii) spread cell surface area (mean ± standard error of mean [SEM]) for both mouse and human platelets indicates a dose‐dependent inhibition of platelet spreading with almost complete spreading blockade at 5 µmol/L SMIFH2. For measurements, N = 3 experiments with each replicate comprising between 150 and 300 platelets per treatment. Live spreading analysis of (C) mouse and (D) human platelets spread on fibrinogen ± 2 or 5 µmol/L SMIFH2. The spread surface area of the platelets was measured at various times after first appearance of the platelet in the imaging field of view and are displayed as mean ± SEM. N = 5 platelets for each treatment. *P < 0.05; **P < 0.01

In mouse platelets treated with increasing concentrations of SMIFH2, platelet spreading on fibrinogen is significantly reduced (Figure S1C in supporting information). Control samples have more than 80% of platelets displaying a nodule/filopodia phenotype, characteristic of mouse platelets spreading on fibrinogen.^19^ With increasing concentrations of SMIFH2 this decreases so that at 5 µmol/L approximately 90% of platelets are unspread (Figure 1Ai). Accordingly, platelet surface area was significantly reduced at both 4 µmol/L (*P* = .027) and 5 µmol/L (*P* = .013) SMIFH2 compared to controls (Figure 1Aii).

### 3.3 Human platelets

A similar effect on spreading is seen in human platelets to that observed in mouse platelets in that spreading is reduced (Figure S1D). Control samples show that approximately 80% of platelets display lamellipodia or are fully spread with an additional ≈15% being at the nodule/filopodia stage. With increasing concentrations of SMIFH2, this decreases so that at 5 µmol/L SMIFH2 approximately 90% of cells are unspread with the remainder displaying occasional small filopodia or poorly formed lamellipodia (Figure 1Bi). Platelet surface area was significantly reduced at 2 µmol/L (*P* = .012), 3 µmol/L (*P* = .003), 4 µmol/L (*P* = .003), and 5 µmol/L (*P* = .001) SMIFH2 (Figure 1Bii).

**Figure 2:**
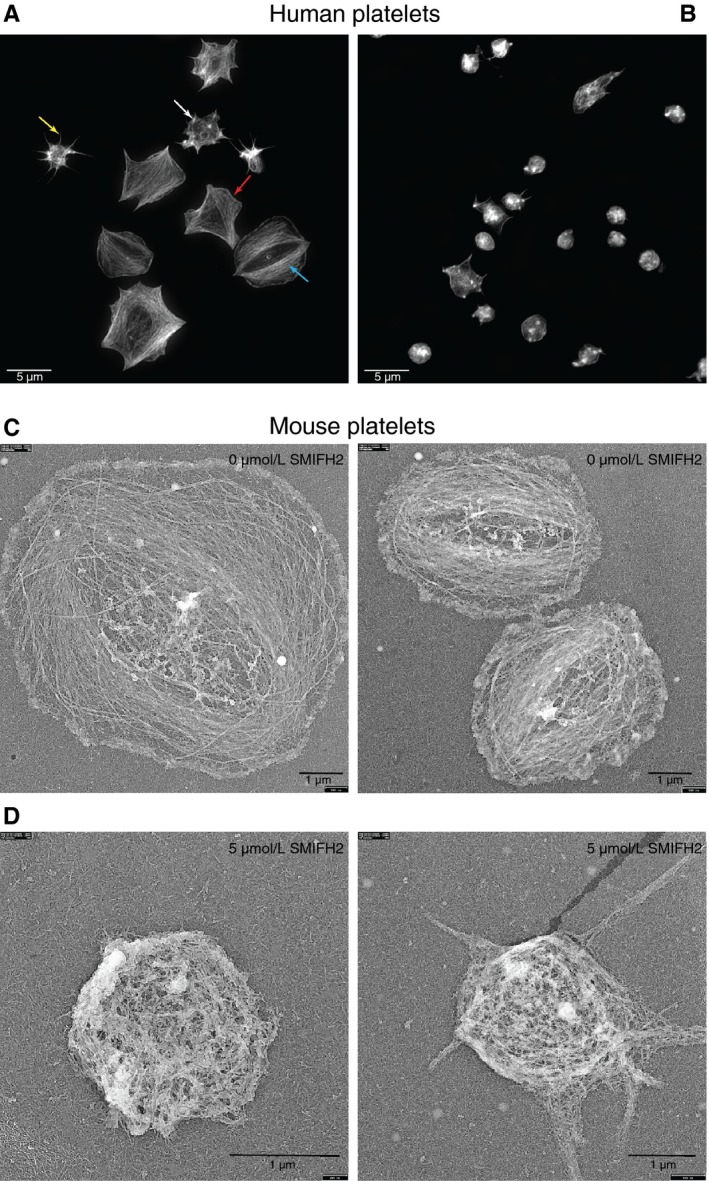
Inhibition of FH2 domains causes disruption to the platelet cytoskeleton. Representative structured illumination microscopy superresolution images of F‐actin in (A) control and (B) 5 µmol/L SMIFH2 treated human platelets spread on fibrinogen (max intensity Z projection, 0.1 µm step size; scale bars = 5 µm) showing filopodia (yellow arrow), lamellipodia (red arrow), actin nodule (white arrow), and stress fibers (blue arrow), which are largely lost in the treated platelets. Platinum replica electron micrographs of (C) control and (D) 5 µmol/L SMIFH2 treated mouse platelets spread on fibrinogen + thrombin showing inability of platelets to spread and reorganize their cytoskeleton (Scale bars = 1 µm)

To test the effect of formin inhibition on the F‐actin content of platelets after activation, human platelets were incubated with SMIFH2 and F‐actin content measured by flow cytometry. Data indicate that SMIFH2 reduced, but did not completely abrogate F‐actin polymerization after activation of platelets with thrombin (Figure S2 in supporting information). To visualize these effects, staining of human platelet F‐actin with Alexa488‐phalloidin and imaging using SR‐SIM reveals that SMIFH2 causes disruption to F‐actin organization. Figure 2A shows control platelets at various stages of spreading displaying characteristic F‐actin organization including nodules, filopodia, lamellipodia, and stress fibers. Pretreatment with SMIFH2 prior to spreading causes disruption to this organization so that many platelets display poorly organized F‐actin and platelets fail to spread properly (Figure 2B). Tubulin organization is also disrupted; at 5 µmol/L SMIFH2 platelets show neither microtubule coils, nor spread cell microtubule networks (Figure S1D). To establish whether this effect was restricted to platelets spreading on fibrinogen, we also tested the effect of SMIFH2 on platelets spreading on fibrinogen following pre‐activation with thrombin (0.1 U/mL) and on collagen‐coated surfaces. In the presence of thrombin, spreading is completely inhibited by 5 µmol/L SMIFH2 (surface area *P* = .0001; Figure S3A in supporting information). On collagen surfaces SMIFH2 completely blocked spreading in both mouse and human platelets at 2 µmol/L (Figure S3B,C). To further visualize the cytoskeleton, mouse platelets treated with 5 µmol/L SMIFH2 or dimethyl sulfoxide (DMSO) were imaged using electron microscopy. In control‐treated cells, the actin formed the characteristic pattern for fully spread platelets (Figure 2C), whereas in platelets treated with SMIFH2, the majority of platelets failed to spread, displayed poorly organized actin, and microtubule coils (Figure 2D). To confirm that the observed effects were not due to nonspecific inhibition of platelet function, human platelets pretreated with SMIFH2 were tested for aggregation and secretion responses to collagen and thrombin. No significant difference was observed for platelets treated with 5 µmol/L SMIFH2 compared to control (Figure S4A‐D in supporting information). We also tested α‐granule secretion and integrin activation in platelets treated with 5 µmol/L SMIFH2 by flow cytometry and although small reductions were observed, platelets were still able to express P‐selectin on their surfaces and to bind fibrinogen (Figure S4E,F). Together these data indicate that the observed effects on spreading are not due to nonspecific inhibition on platelet signalling or due to death of the platelets.

### 3.4 Live platelet spreading

To determine the effect of FH2 inhibition on cytoskeletal and spreading dynamics, platelet spreading on fibrinogen was followed in real time using differential interference contrast (DIC) microscopy for both human and mouse platelets and spread platelet area measured at a range of time points in an approach similar to that of Paknikar et al.^20^ In control mouse platelets, mean surfaces area remained fairly constant for the first 60 seconds after attachment as the platelets began spreading and extended and retracted filopodia. From about 60 seconds onward, the platelets started to form lamellipodia and increased in size (Figure 1C). A similar pattern was observed for control human platelets, although the formation of lamellipodia happened faster than in mouse platelets (Figure 1D). In the presence of 5 µmol/L SMIFH2, both mouse and human platelets failed to spread and remained in this state for the duration of the imaging (Figure 1C,D). For platelets treated with a lower concentration of SMIFH2, the spreading was reduced compared to controls (Figure 1C,D) indicating that at 2 µmol/L SMIFH2, there is still enough FH2‐mediated actin polymerization for limited platelet spreading.

**Figure 3:**
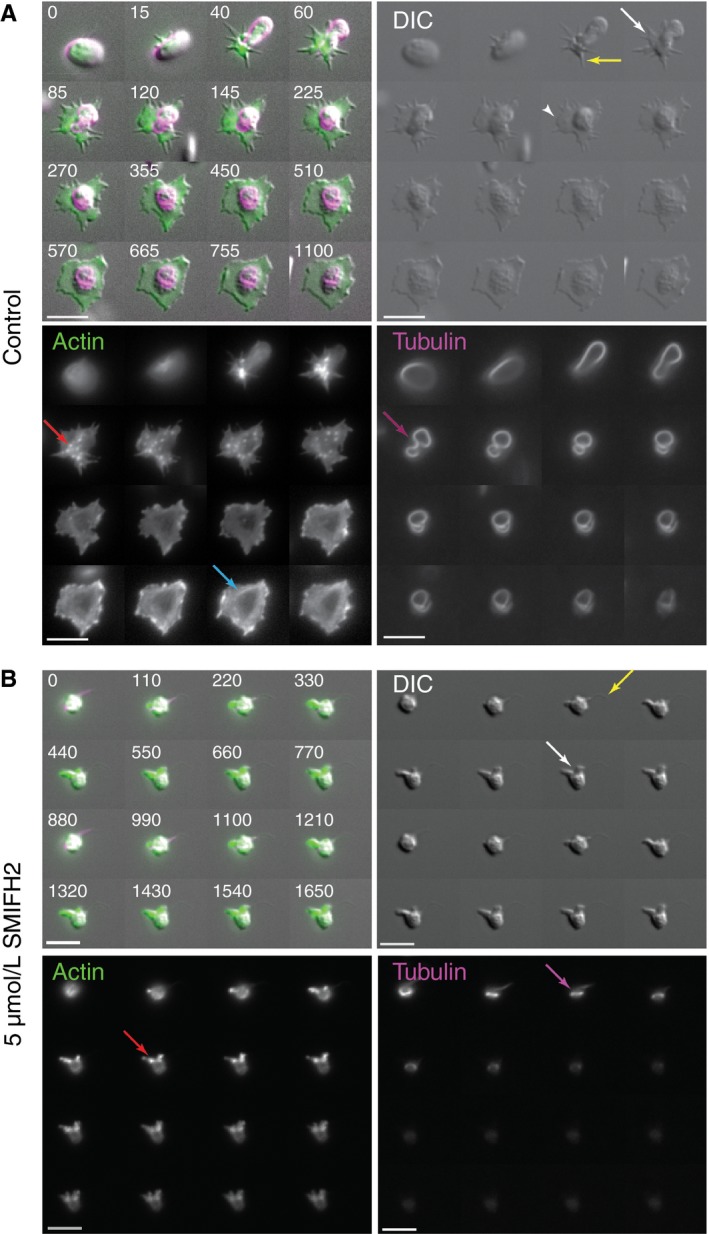
Inhibition of FH2 domains disrupts actin and microtubule dynamics. Representative time‐lapse images from (A) control and (B) 5 µmol/L SMIFH2 treated Lifeact‐GFP mouse platelets spreading on fibrinogen showing effect of FH2 inhibition on morphology (upper right), F‐actin organization (lower left), and tubulin ring organization (lower right). Scale bar = 5 µm. Time in merged images is in seconds. (See also Videos [Link], [Link], [Link], [Link].) Arrows in (A) indicate filopodia (yellow), lamellipodia (white), actin nodules (red), dynamic tubulin rings (magenta), and stress fibers (blue). Equivalent color arrows in (B) highlight where these structures are disrupted. Images are the same focal plane recorded over time

To investigate this effect in more detail, platelets from the Lifeact‐GFP mouse were loaded with SiR‐Tubulin^21^ to allow visualization of the F‐actin and microtubule dynamics. Platelets were then allowed to spread on fibrinogen‐coated coverslips in the presence or absence of 5 µmol/L SMIFH2. Still images of representative platelets can be found in Figure 3A,B. Example videos can be found in Videos [Link], [Link], [Link], [Link] in supporting information. In control cells, the characteristic spreading stages are observed including the generation of filopodia, actin nodules, lamellipodia, and stress fibers as well as the twisting and contraction of the microtubule coil (Figure 3A). In platelets pretreated with 5 µmol/L SMIFH2, spreading dynamics and cytoskeletal organization are completely disrupted. Occasional small protrusions can be observed in treated platelets; however, these seemed to form by blebbing rather than by the conventional actin‐mediated extension. No evidence of “normal” filopodia or lamellipodia formation was observed. Actin nodules appeared, but only one or two were seen per platelet and these did not turn over as observed in control platelets; in addition, the platelets did not form stress fibers. Tubulin dynamics were also disrupted in SMIFH2‐treated cells; upon adhesion/activation, tubulin rings were less obvious than in control platelets and the characteristic twisting and coiling of the ring was not observed. The tubulin ring also seemed to depolymerize much more quickly than in control cells, which often displayed a remnant ring even when fully spread (Figure 3A,B; Videos [Link], [Link], [Link], [Link]).

**Figure 4:**
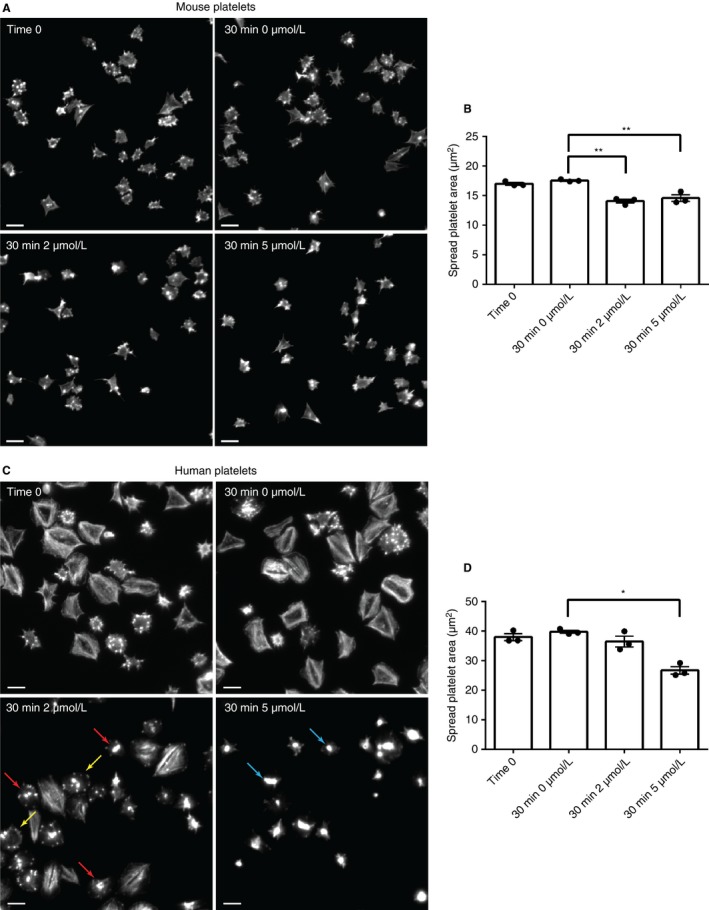
Continued FH2 activity is required to maintain platelet spreading. Representative images of (A) mouse and (C) human platelets spread on fibrinogen for 45 minutes, prior to incubation with 0, 2, or 5 µmol/L SMIFH2 for 30 minutes and staining for F‐actin. Red arrows highlight the loss of stress fibers and the foci of F‐actin at the cell periphery (yellow arrows). Blue arrows highlight the bright foci of F‐actin that accumulated at the center of the platelets (max intensity Z projections, 0.1 µm step size; scale bars = 5 µm). Analysis of spread platelet surface area (mean ± standard deviation) indicates a significant decrease in platelet area upon treatment with SMIFH2 in both (B) mouse and (D) human platelets. N = 3 experiments with each replicate comprising between 150 and 300 platelets per treatment. *P < 0.05; **P < 0.01

The above data indicate that platelet spreading requires formin proteins, as global inhibition of FH2 domains blocks platelet spreading. To establish if continued formin activity is required after platelet spreading, mouse or human platelets were allowed to spread for 45 minutes on fibrinogen before addition of SMIFH2 for 30 minutes (Figure 4). In both human (Figure 4C) and mouse (Figure 4A) platelets, incubation with DMSO vehicle control for 30 minutes had no effect on platelet spreading. However, in human platelets incubated with 2 µmol/L SMIFH2, many platelets displayed no stress fibers and lamellipodia began to retract leaving foci of F‐actin at the cell periphery. Treatment with 5 µmol/L SMIFH2 caused the loss of stress fibers in all platelets and the lamellipodia of most cells to retract. Bright foci of F‐actin were observed at the center of the platelets. This was reflected by a significant reduction in spread platelet area (*P* = .04; Figure 4D). A similar situation was observed in mouse platelets; however, the morphological changes were less clear due to the reduced spreading of mouse platelets on fibrinogen (Figure 4C). Despite this, a significant reduction in spread platelet area (2 µmol/L, *P* = .006; 5 µmol/L, *P* = .008) was observed (Figure 4B). These data indicate that continued formin activity is required once platelets have spread to maintain stress fibers and lamellipodia and hence spreading. Taken together these data show that global inhibition of formin proteins in mouse and human platelets disrupts actin and tubulin dynamics and prevents platelet spreading and that continued formin activity is required to maintain this spread morphology.

### 3.5 Flow data

To establish the effect of inhibiting formin activity on platelet thrombus formation under shear conditions, whole human blood was treated with SMIFH2 prior to flow assays. Surprisingly, no effect of inhibition of FH2 domains was observed on the formation of platelet thrombi as measured by surface area coverage or by monitoring the change in surface area coverage over time (Figure S5A,B, Video S5 in supporting information). In addition, no effect was seen when the concentration of SMIFH2 was increased to 50 µmol/L. To test the hypothesis that the inhibitor was being strongly bound by plasma proteins, washed platelets and platelet‐rich plasma (PRP) were treated with either 0, 5, or 50 µmol/L SMIFH2 before spreading on fibrinogen and staining for F‐actin. The effect of SMIFH2 was completely abrogated in PRP, even when the concentration of inhibitor was increased 10 times (Figure S5C).

As we have demonstrated that continued formin activity is required for maintenance of platelet spreading (Figure 4), we performed experiments in which thrombi were allowed to form for 10 minutes and then were washed for 20 minutes in buffer containing 0, 5, or 25 µmol/L SMIFH2. No effect of formin inhibition was observed on the size of these preformed aggregates when measured as total surface area coverage at the end of the experiment (Figure S5D) or when assessing the change in individual thrombi size pre‐ and postwashing (Figure S5E).

### 3.6 Effect of formin inhibition on the resting platelet cytoskeleton

**Figure 5:**
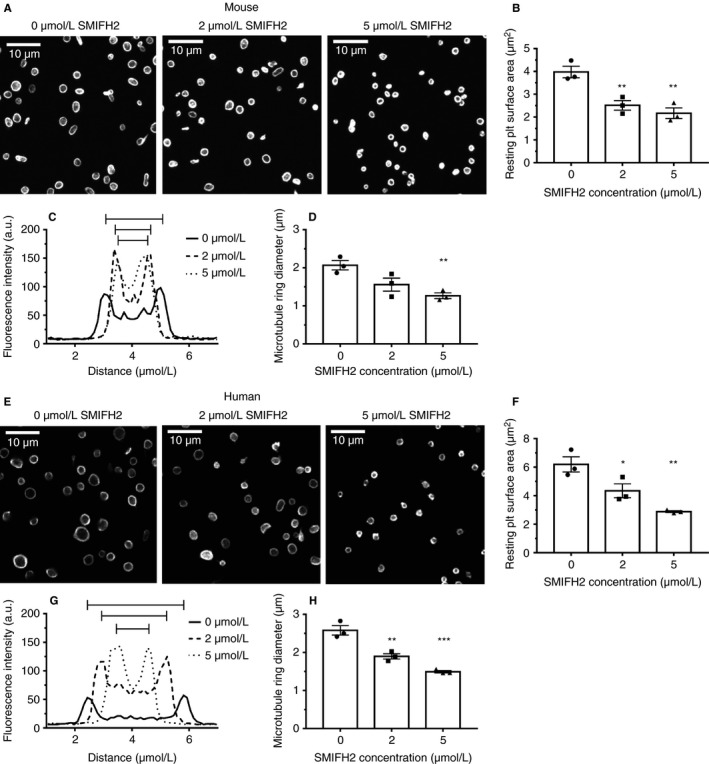
Inhibition of FH2 domains reduces resting platelet size. Representative confocal images of (A) mouse and (E) human resting platelets incubated with SMIFH2 for 3 hours (max intensity Z projections, 0.2 µm step size; scale bars = 10 µm). Analysis of resting platelet surface area indicates a significant decrease in platelet size upon treatment with SMIFH2 in both (B) mouse and (F) human platelets. Analysis of the microtubule rings of these platelets indicates that the microtubule ring remains intact upon SMIFH2 treatment, but their average diameter is reduced in both mouse (C & D) and human (G & H) platelets. For all plots data are mean ± standard error of mean. For measurements, N = 3 experiments with each replicate comprising between 150 and 300 platelets per treatment. *P < 0.05; **P < 0.01; ***P < 0.005

Human platelets with a constitutively active mDia1^17^ demonstrate a macrothrombocytopenia. To establish if the FH2 domain of formin proteins plays a role in this size increase, resting mouse and human platelets were treated with SMIFH2 before fixation and staining for the microtubule coil. Inhibition of FH2 domains in both mouse (Figure 5A,B) and human (Figure 5E,F) platelets caused a significant, dose‐dependent decrease in the surface area of resting platelets (Mouse 2 µmol/L, *P* = .007; 5 µmol/L, *P* = .003; Human—2 µmol/L, *P* = .03; 5 µmol/L, *P* = .002). However, in both cases the reduction in size is not caused by depolymerization of the microtubule coil; SMIFH2‐treated platelets still display a microtubule coil, but one which is significantly smaller in diameter than in control cells (Mouse—5 µmol/L, *P* = .009; Human—2 µmol/L, *P* = .003; 5 µmol/L, *P* = .0002; Figure 5C, 5,G,H).

**Figure 6:**
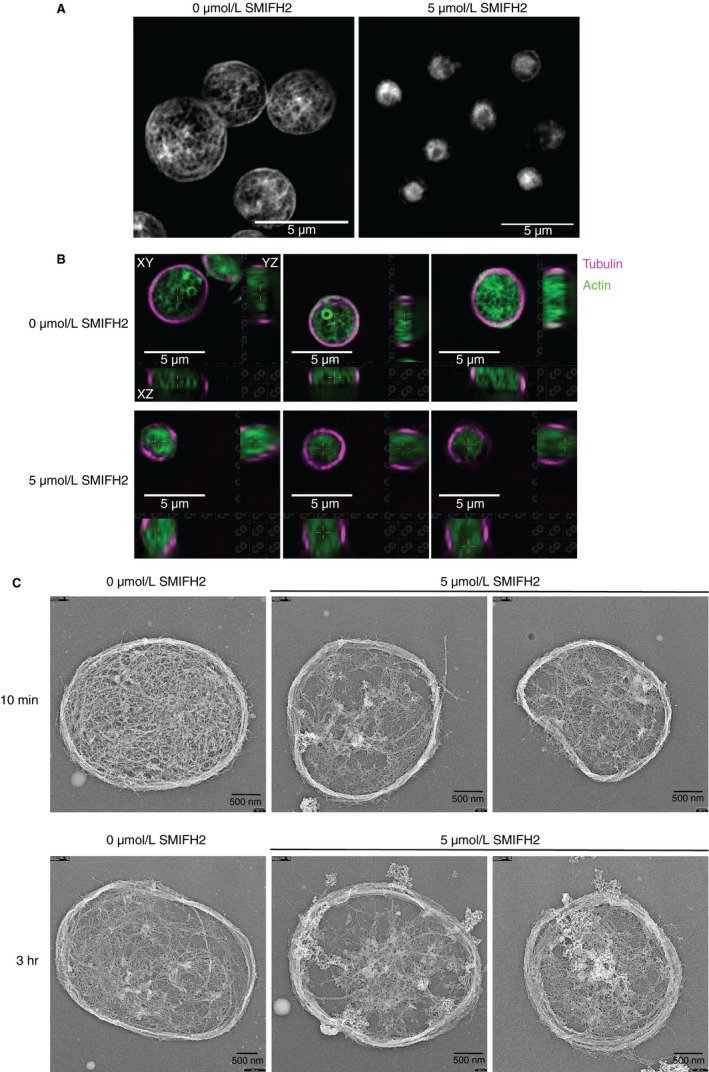
Disruption of resting platelet F‐actin by SMIFH2. A, Representative superresolution structured illumination microscopy (SR‐SIM)images of resting human platelets treated with 0 or 5 µmol/L SMIFH2 and stained for F‐actin (max intensity Z projections, 0.1 µmol/L step size; scale bars = 5 µm). See also Videos S6 and S7. B, Orthogonal projections of SR‐SIM images of resting human platelets treated with 0 or 5 µmol/L SMIFH2 and stained for F‐actin (green) and α‐tubulin (magenta). Control platelets demonstrate the characteristic discoid shape (upper panel), which is lost upon SMIFH2 treatment (lower panel). Instead, platelets become rounded, losing the F‐actin organization, yet they retain their microtubule coil (scale bars = 5 µm). C, Example platinum replicas of resting mouse platelets treated with 0 or 5 µmol/L SMIFH2 for 10 minutes (upper panel) or 3 hours (lower panel). Both the F‐actin network and the microtubule coil are perturbed and are less well defined as in the control. Treated platelets are also smaller in size and appear rounder with thickened microtubule coils than the control (scale bars = 1 µm)

In addition to the change in diameter of resting platelets, superresolution structured illumination microscopy (SR‐SIM) imaging of the resting actin cytoskeleton demonstrates that inhibition of FH2 domains causes the loss of the F‐actin network identified in control resting platelets (Figure 6A; Videos S6 and S7 in supporting information). This is accompanied by rounding of the cells (as can be observed by visualizing orthogonal projections of SR‐SIM z stacks), compared to control platelets, which retain their discoid shape (Figure 6B). To further establish the effect of FH2 inhibition, the cytoskeleton of resting platelets treated with either DMSO or 5 µmol/L SMIFH2 was visualized by electron microscopy (Figure 6C). Platelets treated with inhibitor for 10 minutes were smaller than controls and the extensive, filamentous F‐actin network of the platelets was disrupted (Figure 6C, upper panel). This effect was even more prominent after 3 hours of SMIFH2 treatment and in addition, resting platelets were considerably smaller and displayed microtubule coils which seemed to display more coils (Figure 6C, lower panel). These data therefore indicate that the FH2 domains of formin proteins are important for the maintenance of resting platelet size and cytoskeletal organization.

### 3.7 Effect of FH2 inhibition on microtubule post‐translational modifications

**Figure 7:**
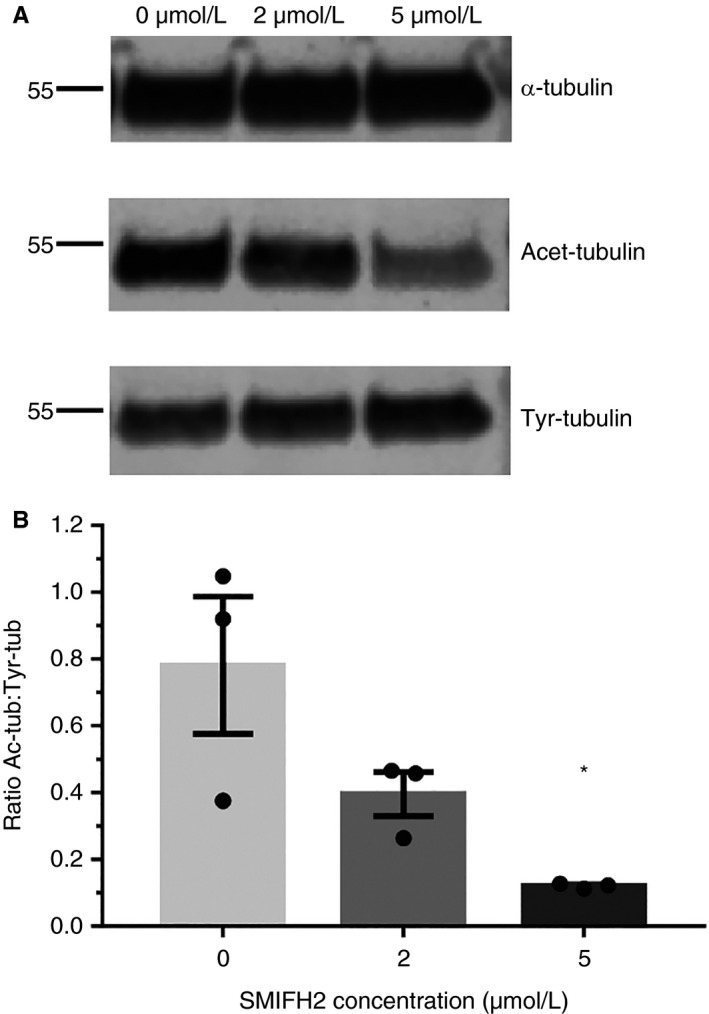
Inhibition of FH2 domains affects microtubule stability. A, Western blots of resting human platelet lysates probed for *α*‐tubulin and its post‐translational modifications, acetylation and tyrosination. B, Quantification of band intensities shows a reduction in the ratio of acetylated:tyrosinated tubulin in SMIFH2‐treated cells (mean ± standard error of mean). N = 3. *P < 0.05

Formins can bind to microtubules via their FH2 domain and have been shown to regulate microtubule dynamics independently of effects on actin dynamics.^22^ Formins have been implicated in the acetylation of microtubules, a modification which, in combination with detyrosination, marks stable microtubules.^23^ We therefore tested the effect of formin FH2 domain inhibition on microtubule post‐translational modification (PTM) in platelets. In resting human platelets treated with SMIFH2, a reduction was observed in the acetylation status and a small increase in the tyrosination status of *α*‐tubulin (Figure 7A; Figure S6 in supporting information). This resulted in a decrease in the acetylation:tyrosination status of tubulin in these platelets (Figure 7B), which was significant at 5 µmol/L SMIFH2 (5 µmol/L, *P* = .017). These data indicate that PTMs of *α*‐tubulin in platelets are regulated, at least in part, by the action of the formin FH2 domain.

## 4 DISCUSSION

The data presented in this study indicate that the FH2 domain of formin proteins is critical for normal cytoskeletal organization and dynamics of platelets, as a dose‐dependent inhibition of platelet spreading by SMIFH2 was observed in both human and mouse platelets. While the data are generated using a single pharmacological inhibitor of formin function, it is well established that the FH2 domain is highly conserved across species and is absolutely required for the actin polymerization action of formin proteins.^4^ Thus, the dose‐dependent effects of SMIFH2 on platelet spreading can be largely explained as a result of decreased actin polymerization. The fact that SMIFH2 inhibition only partially blocks F‐actin polymerization in platelets activated in suspension (Figure S2) shows that other mechanisms (ie, Arp2/3‐mediated polymerization) remain intact and contribute to the F‐actin polymerization seen in activated platelets. Thus, formin‐mediated F‐actin may provide filaments from which Arp2/3 nucleation can occur to drive lamellipodia formation in adherent cells. This interaction between formin‐ and Arp2/3‐mediated processes has been seen in other cell types^24^ and thus may underlie the strong effect on platelet spreading seen here. It is interesting to note that platelets treated with the Arp2/3 inhibitor CK666 also show a complete block in platelet spreading,^25^ indicating that both formin‐ and Arp2/3‐mediated nucleation are required for proper spreading. However, data from models of reduced Arp2/3 complex expression show that full spreading is lost, but platelets are still able to form filopodia,^26^, ^27^ presumably via the action of formins and the presence of preformed actin filaments. The observation that adding SMIFH2 to platelets that had already spread resulted in the loss of stress fibers and lamellipodia collapse (Figure 4) would seem to support the hypothesis that lamellipodia formation and persistence requires formin‐mediated actin filaments. It is interesting that adding SMIFH2 to preformed platelet thrombi had no effect on their size or stability, even when added at five times the concentration that blocks platelet spreading (Figure S5). Whether this reflects that actin filaments/lamellipodia are not required for aggregate stability at this stage, as has recently been reported by Schurr et al,^28^ is an intriguing possibility.

The possible off‐target and cytotoxic effects of SMIFH2 have been investigated in other studies.^18^ To minimize any possible off‐target effects, lower concentrations and shorter incubation times than those studies were used, which are well below the concentrations reported to have effects on p53 function and Golgi organization.^29^ Furthermore, platelets treated with 5 µmol/L SMIFH2 retain normal aggregation and secretion responses indicating that the platelet are viable. Therefore, it is reasonable to conclude that formin‐mediated actin polymerization is a key component of platelet spreading in response to a range of platelet agonists, a critical step in the initial adhesion and spreading of the first platelets to arrive at the site of vascular damage.

In addition to the disruption of platelet actin dynamics, changes in microtubule organization and dynamics were observed. Microtubule coils in platelets at early stages of spreading were disrupted and spread platelet microtubule networks failed to form. Furthermore, the dynamics of microtubules were affected with observed microtubule coils failing to undergo the characteristic twisting of control platelets (as observed by Diagouraga et al^30^) and appearing to depolymerize more rapidly than in controls. Thus, it would appear that there is an effect of SMIFH2 on microtubules in addition to the inhibition of actin polymerization. There is substantial evidence that formins can regulate microtubule dynamics, both via direct binding of formins to tubulin, as well as via interaction with microtubule associated proteins (reviewed by Zuidscherwoude et al[7]) and that SMIFH2 can disrupt microtubule dynamics.^29^ However, whether the effects observed here on spread platelet microtubule dynamics are due to blocking a direct interaction of FH2/formins with microtubules, or through indirect effects on actin polymerization, is unclear. There is evidence that formins can stabilize microtubules independently of their effect on actin^22^ and that competition for FH2 domains between actin filaments and microtubules regulates actin and microtubule interactions.^31^ Regardless of the mechanism, such studies highlight the need for orchestration of the actin and microtubule cytoskeletons for proper platelet function, which is clearly lacking in platelets treated with SMIFH2.

The importance of this cytoskeletal orchestration is supported by a key observation in this work, namely the effect of FH2 domain inhibition on the resting platelet. Imaging of the actin cytoskeleton of resting platelets by SIM and electron microscopy (EM) imaging shows that inhibition of FH2 domains results in the loss of the organized F‐actin network seen in resting platelets and subsequent loss of the platelet discoid shape. Furthermore, it is well characterized that depolymerization of the microtubule coil, as in the case of treatment with microtubule depolymerizing agents or by chilling of platelets, results in a reduced platelet size. However, here the decrease in size was not accompanied by substantial microtubule depolymerization, rather the microtubule coil was intact, but smaller in diameter as if it had coiled in on itself. This hypothesis is supported by evidence from resting platelet electron micrographs which shows thicker microtubule coils. To establish a possible mechanism by which this might occur, we looked at the PTM status of tubulin in resting platelets when incubated with SMIFH2, and determined that the ratio of acetylated:tyrosinated tubulin is reduced when FH2 domains are inhibited. Thurston et al^23^ demonstrated that microtubule acetylation was a general feature of FH2 domains, but it is unclear whether this is due to activation of a specific tubulin acetyltransferase (TAT) or inhibition of histone deacetylases (eg, HDAC6).^32^ Recent evidence suggests that formins play a dual role by stabilizing microtubules to allow tubulin acetylation as well as by increasing the transcription of TAT1,^33^, ^34^ although clearly the latter will not be occurring in platelets.

Tubulin PTMs have been shown to be important in regulating both microtubule stability and their interaction with microtubule associated proteins and are part of a tubulin code that regulates cytoskeletal dynamics.^32^, ^35^, ^36^ Furthermore, tubulin PTMs, including acetylation, polyglutamylation, and polyglycylation have been shown to be important in platelets and megakaryocytes.^37^, ^38^ The changes reported here could affect the platelet microtubule coil in a number of ways: (a) tubulin acetylation is associated with the protection of microtubules from mechanical stress,^36^ (b) increased tyrosination is associated with more dynamic microtubules^39^, and (c) increased tyrosination is associated with increased kinesin motor activity.^40^ Together, these may explain the changes in microtubules observed in resting platelets upon FH2 inhibition. Increased kinesin motor activity and more dynamic microtubules, combined with a reduction in microtubule–actinomyosin cross‐linking can cause the microtubule coil to tighten up and decrease in size, reducing the size of the resting platelets. In addition, decreased acetylation of microtubules makes them more prone to mechanical stress and may be why the microtubule coil depolymerizes more quickly in spreading platelets treated with SMIFH2. Together these data support the idea that a balance of dynamic and stable microtubules in the resting platelet microtubule coil is required as proposed by Patel‐Hett et al.^39^

There is a precedent in the literature regarding formin proteins, platelets, and microtubule PTM status. Both Pan et al and Stritt et al have reported altered microtubule PTM in models of platelet and megakaryocyte formin disruption.^15^, ^17^ Although the genetic approaches employed in those studies resulted in a loss or reduction of protein expression, we have demonstrated here that blocking the action of platelet formin FH2 domains alone is sufficient to disrupt microtubule PTMs and subsequent function. Therefore, although we cannot shed direct light on the mechanism of PTM regulation, we demonstrate the importance of a properly regulated cytoskeleton for platelet function.

**Figure 8:**
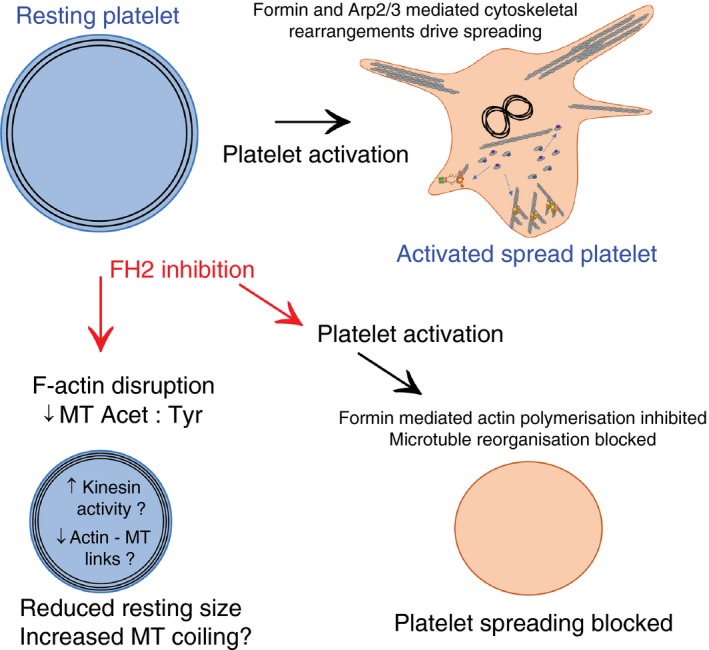
Proposed model of how SMIFH2 disrupts platelet function. A, The well‐characterized actin and microtubule organization of the resting platelet is rapidly modified upon activation to allow platelet shape change and spreading. B, We propose that inhibition of FH2 domains affects both the cytoskeleton of the resting platelet (via changes in microtubule post‐translational modifications and loss of the organized F‐actin network) and in activated platelets (by preventing formin‐mediated F‐actin polymerization and MT reorganization)

In conclusion, we demonstrate that the FH2 domain of formin proteins plays a key role in the organization and dynamics of the platelet actin and tubulin cytoskeletons (Figure 8). The fact that mDia1 knockout platelets show no spreading phenotype would seem to support the hypothesis that multiple formin proteins can fulfil this role during platelet activation and thus the challenge is now to identify the contribution that each of the platelet‐expressed formin proteins makes to this process.

## CONFLICTS OF INTEREST

The authors declare no competing financial interests.

## AUTHOR CONTRIBUTIONS

Concept and design: S.G. Thomas; analysis and interpretation: H.L.H. Green, M. Zuidscherwoude, F. Alenazy, C.W. Smith, M. Bender, S.G. Thomas; critical writing and revision: H.L.H. Green, M. Zuidscherwoude, S.G. Thomas; final approval: H.L.H. Green, M. Zuidscherwoude, F. Alenazy, C.W. Smith, M. Bender, S.G. Thomas.
