# Supplementary material for: SMIFH2 inhibition of platelets demonstrates a critical role for formin proteins in platelet cytoskeletal dynamics
Source: J Thromb Haemost. 2020 Feb 17;18(4):955–67. doi: 10.1111/jth.14735 (PMC7186844; doi:10.1111/jth.14735)

SMIFH2 inhibition of platelets demonstrates a critical role for formin proteins in platelet cytoskeletal dynamics

Hannah L.H. Green [*,†,¶], Malou Zuidscherwoude [*,‡,¶], Fawaz Alenazy [*], Christopher W. Smith [*], Markus Bender [§] & Steven G. Thomas [*,‡,**]

[*] Institute of Cardiovascular Sciences, University of Birmingham, Edgbaston, Birmingham, UK.
[†] Current Address: School of Cardiovascular Medicine & Sciences, BHF Centre of Research Excellence, King's College London, London, UK.
[‡]Centre of Membrane Proteins and Receptors (COMPARE), University of Birmingham and University of Nottingham, Midlands, UK
[§] Rudolf Virchow Center, University of Würzburg, Würzburg, Germany.
[¶] These authors contributed equally to the work
[**] Author for correspondence

Supplementary Information

Includes

- Detailed methods
- Supplementary video legends
- Supplementary figures and figure legends

Methods

Mice

C57Bl/6 or Lifeact-GFP [1] mice were maintained in IVCs under 12 h light/dark cycle at a constant temperature of 20 ºC with food and water given *ad libitum* at BMSU, University of Birmingham, UK. All experiments were performed in accordance with UK laws (Animals in Scientific Procedures Act 1986) with approval of local ethics committee (Birmingham Animal Welfare and Ethical Review Board – AWERB) under a Home Office approved project licence.

Platelet preparation

Human washed platelets were prepared from blood samples donated by healthy, consenting volunteers (local ethical review no: ERN-11-0175). Blood was drawn via venepuncture into sodium citrate as the anticoagulant and then acid/citrate/dextrose (ACD) was added to 10% (v:v). Blood was centrifuged at 200 x g for 20 min. Platelet rich plasma (PRP) was obtained and then centrifuged at 1000 x g for 10 min in the presence of 0.1 μg ml^{-1} prostacyclin (Cayman Chemicals). Plasma was removed and the platelet pellet was resuspended in modified Tyrode's buffer (134mM NaCl, 0.34mM Na_2HPO_4, 2.9mM KCl, 12mM $NaHCO_3$, 20mM HEPES, 5mM glucose, 1mM $MgCl_2$; pH 7.3) containing ACD and 0.1 μg ml^{-1} prostacyclin before being centrifuged for 10 min at 1000 x g. The washed platelet pellet was resuspended in modified Tyrode's buffer, left to rest for 30 min and the platelet count determined using a Coulter Counter (Beckman Coulter). Platelet count was adjusted as required using modified Tyrode's buffer.

Mouse washed platelets were prepared from blood drawn from the vena cava of CO_2 narcosed mice directly into 100 μl ACD. PRP was obtained by centrifugation at 200 x g for 6 min. Washed platelets were prepared via centrifugation of PRP at 1000 x g in the presence of prostacyclin (0.1 μg ml^{-1}) for 6 min. The pellet was resuspended in modified Tyrode's buffer and counted as per human platelets.

Platelet spreading

For fixed cell immunofluorescence imaging, platelets were diluted to 2 x 10^{7} ml^{-1} in modified Tyrode's buffer and allowed to spread on fibrinogen (100 μg ml^{-1}) (Enzyme Research) or 95%

Type I, 5% Type III (Horm) collagen (10 μg ml^{-1}) (Takeda/Nycomed) coated coverslips for 45 min at 37 °C, 5 % CO_2. Where indicated, platelets were preactivated with 0.1 U ml^{-1} thrombin (Sigma) prior to plating on fibrinogen coverslips. For inhibitor studies, SMIFH2 (Merck Millipore) was added to the cells at the required concentration for 10 min at room temperature prior to spreading and was present throughout spreading. Control samples were treated with DMSO at an equivalent volume to that used in SMIFH2 treated samples and the maximum concentration of DMSO used was 0.5 % (v/v). following spreading, coverslips were washed once in PBS to remove non-adhered platelets then fixed in 10 % neutral buffered formalin (Sigma-Aldrich) for 10 min. For experiments to establish the effect of adding the inhibitor to spread platelets, mouse and human platelets were allowed to spread on fibrinogen coated coverslips for 45 min as described above. Non-adherent platelets were removed and the coverslips washed once with modified Tyrode's buffer at 37 °C before incubating in Tyrode's containing 2 or 5 μM SMIFH2 or DMSO vehicle control for 30 min. Coverslips were then washed and fixed as described above.

For live cell imaging experiments, washed human or Lifeact-GFP mouse platelets were prepared as above and diluted to 2 x 10^8 ml^{-1} before being incubated with 1 μM SiR-tubulin (Spirochrome) at 37 °C for 1 hour. Platelets were diluted to 4 x 10^7 ml^{-1} and incubated with SMIFH2 at the required concentration before adding to fibrinogen coated glass bottomed dishes (MatTek) and imaging.

Resting platelets

To test the effect of FH2 inhibition on resting platelet, washed human and mouse platelets at 4 x 10^7 ml^{-1} were incubated with SMIFH2 at 37 °C for 3 hours before fixation in pre-warmed formalin for 10 min. Fixed platelets were added to a 24-well plate containing poly-L-lysine coated glass coverslips and centrifuged to immobilise platelets before washing and staining as described below.

Immunolabelling

Following PBS washes cells were permeabilised with 0.1 % (v/v) Triton X-100 for 5 min and then washed in PBS and blocked for 30 min in block buffer (1 % BSA, 2 % goat serum in PBS). Tubulin was immunolabelled with 2 μg ml^{-1} anti-α-tubulin (Clone DM1A, Sigma-Aldrich) diluted

in block buffer for 1 hr at room temperature. Tubulin was secondary labelled with 4 μg ml^{-1} anti mouse-Alexa647 and F-actin was labelled with 13 nM phalloidin-Alexa488 (Thermo-Fisher). For epifluorescence or confocal imaging, cells were washed and mounted in Hydromount (National Diagnostics) prior to imaging. For SR-SIM imaging, cells were mounted in Prolong Glass mounting medium (ThermoFisher).

Visualisation of the platelet cytoskeleton using electron microscopy

To prepare the cytoskeleton, platelets were allowed to adhere to poly-L-lysine coverslips or spread on fibrinogen coverslips for 45 min and then incubated with PHEM buffer supplemented with 0.75% Triton X-100, 1 µM phalloidin, 1 µM taxol and 0.1% glutaraldehyde. After washing with PHEM buffer containing 0.1 µM phalloidin and taxol, adherent platelets were fixed for 10 min in PHEM buffer supplemented with 1% glutaraldehyde.

Subsequently, fixed cells were incubated with 0.1% tannic acid and 0.2% uranyl acetate for 20 minutes each. Dehydration was performed by transferring samples through graded acetone and then critical point drying was performed in a Leica EM CPD300. Samples were finally coated with 1.2 nm of platinum with rotation at 45°C and 3 nm of carbon at 90°C without rotation under high vacuum in a Leica EM ACE600. Replicas were floated, picked up on formvar-carbon-coated grids and examined using a JEOL JEM-2100.

SDS-PAGE and Western blotting

Lysates from platelets incubated for 3 hours with 0, 2 or 5 μM SMIFH2 were prepared from platelets at 5 x 10^8 ml^{-1} in an equal volume of 2x lysis buffer on ice for 10 minutes. Lysates were mixed with sample buffer and boiled for 5 minutes before cooling. Samples and protein marker were loaded into wells of pre-cast polyacrylamide gels (Bolt, Invitrogen) and run at 60 V for 15 minutes to stack proteins, followed by 120 V for 1 hour for separation. Proteins were transferred to PVDF membranes for 10 minutes using the Trans-Blot Turbo Transfer System (Bio-rad), then incubated for 1 hour at room temperature in blocking buffer (5 % BSA in TBST, filtered). Blots were incubated with primary antibodies against either α-tubulin (clone DM1A, Sigma-Aldrich), acetylated α-tubulin (Clone 6-11B-1, Cell signalling Technology) or tyrosinated α-tubulin (Clone YL1/2, Merck Millipore) in blocking solution at 4 ^{o}C overnight. Blots were washed 3 times for 10 minutes each in TBST, then incubated for 1 hour with anti-mouse or anti-

rat secondary antibody, diluted 1:10000 in TBST, before a further 3 washes. Using an Odyssey imaging system and Image Studio software (Li-Cor Biosciences), Western blots were exposed for 2-10 minutes and band intensities were quantified. For each antibody, a separate blot was used to avoid stripping and re-probing artefacts.

Platelet aggregation and secretion

Platelet aggregation and dense granule ATP secretion was monitored using 300 μL of washed platelets at 2 x 10^8 mL^{-1}. Stimulation of platelets with thrombin (0.06 U mL^{-1}) or Collagen (4 µg mL^{-1}) was performed in a Lumi-Dual Aggro-meter (Chrono-Log Corporation) with continuous stirring at 1200 rpm at 37 °C. ATP secretion was determined during aggregation using Chrono-Lume reagent (Chrono-Log Corporation). Where inhibitors were used these were added to the platelets for 10 min prior to addition of platelet agonists.

Flow cytometry

Platelet activation in the presence or absence of 5 µM SMIFH2 (for either 10 min or 3 hr) was monitored by measuring P-selectin exposure, fibrinogen binding and F-actin polymerisation following platelet stimulation. For P-selectin and fibrinogen binding, washed platelets at 2 x 10^8 mL^{-1} were stimulated with 0.1 U mL^{-1} thrombin at 37 °C for 2 min. Stimulated platelets were then incubated with either anti-P-Selectin-FITC (Clone AK-4, BD Biosciences) or fibrinogen-Alexa488 (ThermoFisher) for 30 min in the dark at room temperature. Samples were then diluted 5x in PBS and fluorescence intensity measured using an Accuri C6 flow cytometer (BD Biosciences). Initial gating of platelets was performed using Forward Scatter/Side Scatter plots and then the fluorescence intensity (in FL1 channel) of 10,000 platelets in this gate was recorded for each sample. For F-actin measurements, platelets were stimulated as above, but then fixed in 10 % neutral buffered formalin (Sigma-Aldrich) for 10 min before washing 1x in PBS. Fixed platelets were stained in F-actin staining solution (PBS, 0.1% v/v Triton x-100, 0.1mM Phalloidin-488) for 60 min in the dark at room temperature. Samples were then diluted and fluorescence intensity measured as described above. Data were analysed using the Accuri C6 software.

In vitro flow

For *in vitro* flow studies, human blood was drawn into sodium citrate. Whole blood was incubated for 10 min at 37 ºC with DiOC6 (final concentration 2 μM) and either DMSO or SMIFH2 at the required concentration prior to starting the flow experiment. Treated blood was flowed over Horm collagen (50 μg mL^{-1}) coated Ibidi μ-Slide VI 0.1 multi-channel slides (Thistle Scientific) at a shear rate of 1000 s^{-1} for 10 min and thrombi formation was followed by taking a z-stack (41 images, step size 0.5 μm) every 15 seconds for the duration of the experiment using an Evos FL Auto imaging system (Life Technologies). Following completion of the flow, images of the thrombi formed were taken at 10 positions evenly spaced along the chamber for endpoint analysis. For washing experiments, platelet thrombi were allowed to form for 10 min as described above before washing for 20 min in Tyrode's buffer containing either 0, 5 or 25 μM SMIFH2. Z stacks were taken at 10 min and 30 min and the size of thrombi compared.

Microscopy

Images were acquired using an Axio Observer 7 inverted epifluorescence microscope (Carl Zeiss Microscopy) with Definite Focus 2 autofocus, 63x 1.4 NA oil immersion objective lens, Colibri 7 LED illumination source, Hammamatsu Orca Flash 4 V2 sCMOS camera, Filter sets 38 and 50 for Alexa488 and Alexa647 respectively and DIC optics. LED power and exposure time were chosen as appropriate for each set of samples but kept the same within each experiment. Using Zen 2.3 Pro software, five image stacks (step size 0.2 μm) were taken per coverslip for fixed platelet experiments. For live cell experiments, images were taken at 23 ° C for 30 minutes at 5 second intervals using differential interference contrast (DIC) to visualise the extent of platelet spreading and Zeiss triple filter set 105 in combination with 488nm and 647nm LEDs to visualise Lifeact-GFP and SiR-tubulin. For resting human platelet experiments, confocal images were acquired using an SP2 confocal microscope (Leica Microsystems) with a 63x 1.4NA oil immersion objective lens and Ar 488 and HeNe 633 lasers. Five image stacks (step size 0.2 μm) were taken per coverslip using Leica confocal software. For SR-SIM imaging, samples were imaged on a Nikon N-SIM-S microscope with Ti2-E inverted microscope stand, PFS4 focus stability system, 100x 1.49 NA oil immersion objective lens with automatic correction collar adjustment, LU-NV-L laser bed, Hamamatsu Orca Flash 4 V3 sCMOS camera, TI2-FT N-SIM Motorized Filter set, and NIS-AR elements V5 software. Raw images were captured in 3D-SIM

mode (step size 0.1 μm), and final SR-SIM images were processed using the 'Stack Reconstruction' feature of NIS-Elements. Reconstructed images were manually checked for processing artefacts and Fourier transforms of the data were checked to confirm appropriate reconstruction [2].

Image analysis

Post capture image analysis was performed using Fiji [3]. For fixed cell measurements, data are means from three independent experiments, with between 150 and 300 individual platelets analysed for each treatment per experiment. For platelet morphology categorisation measurements, examples of platelets in each category are given in Supp. Fig 1A & B. For live cell spreading analysis, individual platelets were cropped from the field of view to allow synchronisation of spreading analysis. The spread platelet area was measured using ROI manager and Measure functions of Fiji. For live cell measurements data are from means from three independent experiments, with 5 platelets measured for each treatment per experiment.

Images of thrombus formation under flow conditions were analysed in Fiji as follows. Stacks of images (either time series of Z-stack maximum intensity projections, or stacks of endpoint images) were background subtracted, and then converted into binary images using the Otsu threshold function. Measurement of the thrombi in each image was obtained using the Analyse Particles function and overall surface area coverage in each image calculated by summing the individual thrombi. For washing experiments, the total surface area coverage at the end of the washing period was calculated as described above and in addition, the change in size of individual thrombi pre and post washing was calculated.

Statistics

Data analysis was carried out using Microsoft Excel and GraphPad Prism V6. Results are shown either as mean ± SEM or mean ± SD (as indicated in the figure legends) and statistical significance was analysed using One-way ANOVA with Dunnetts multiple comparison tests. P values are given in the text whilst in figures is represented by stars ($P<0.05$ = *; $P<0.01$ = **; $P<0.005$ = ***).

References

1 Riedl J, Flynn KC, Raducanu A, Gärtner F, Beck G, Bösl M, Bradke F, Massberg S, Aszodi A, Sixt M, Wedlich-Söldner R. Lifeact mice for studying F-actin dynamics. *Nature Methods*. 2010; **7**: 168.

2 Poulter NS, Khan AO, Pallini C, Thomas SG. Single-Molecule Localization and Structured Illumination Microscopy of Platelet Proteins. In: Gibbins JM, Mahaut-Smith M, eds. *Platelets and Megakaryocytes : Volume 4, Advanced Protocols and Perspectives*. New York, NY: Springer New York, 2018, 33-54.

3 Schindelin J, Arganda-Carreras I, Frise E, Kaynig V, Longair M, Pietzsch T, Preibisch S, Rueden C, Saalfeld S, Schmid B, Tinevez J-Y, White DJ, Hartenstein V, Eliceiri K, Tomancak P, Cardona A. Fiji: an open-source platform for biological-image analysis. *Nature Methods*. 2012; **9**: 676.

Supplementary Video Legends

Supplementary Video 1

Example time-lapse of control treated mouse platelets spreading on fibrinogen and imaged for F-actin (upper right), microtubules (lower left) and morphology (lower right). A merged imaged is shown in the upper left panel.

Supplementary Video 2

Example time-lapse of control treated mouse platelets spreading on fibrinogen and imaged for F-actin (upper right), microtubules (lower left) and morphology (lower right). A merged imaged is shown in the upper left panel.

Supplementary Video 3

Example time-lapse of 5 μM SMIFH2 treated mouse platelets spreading on fibrinogen and imaged for F-actin (upper right), microtubules (lower left) and morphology (lower right). A merged imaged is shown in the upper left panel.

Supplementary Video 4

Example time-lapse of 5 μM SMIFH2 treated mouse platelets spreading on fibrinogen and imaged for for F-actin (upper right), microtubules (lower left) and morphology (lower right). A merged imaged is shown in the upper left panel.

Supplementary Video 5

Example videos of the effect of SMIFH2 on the formation of platelet thrombi on collagen at shear of 1000 s-1. Time stamp (min:sec). Scale bar = 25 µm.

Supplementary Video 6

Example SR-SIM Z-stack of resting human platelets treated with 0 μM SMIFH2 and imaged for F-actin. Scale bar = 5 µm, Z step size = 0.1 µm.

Supplementary Video 7

Example SR-SIM Z-stack of resting human platelets treated with 5 μM SMIFH2 and imaged for F-actin. Scale bar = 5 µm, Z step size = 0.1 µm.

Supplemental Figure Legends

Supplementary figure 1: Example images showing the categories used to define **A)** mouse and **B)** human platelet morphology in Figure 1. **C & D)** Representative widefield epifluorescence images of mouse **C)** and human **D)** platelets spread on fibrinogen in the presence of 0, 2 or 5 μM SMIFH2. Platelets were stained for F-actin and α-tubulin. Scale bar = 10 μm.

Supplementary figure 2: Effect of SMIFH2 on F-actin polymerisation following platelet activation. **A)** Example flow cytometry traces for platelets treated with 0 or 5 μM SMIFH2 and either stimulated with PBS or thrombin. **B)** Quantification of F-actin polymerisation assays showing effect of SMIFH2.

Supplementary figure 3: A) Effect of SMIFH2 on human platelets spreading on fibrinogen in the presence of 0.1 U mL^{-1} thrombin. Scale bars = 10 μm. **B & C)** Effect of SMIFH2 on spreading of **B)** mouse and **C)** human platelets on collagen. Scale bar = 5 μm.

Supplementary figure 4: Effect of SMIFH2 on aggregation of human platelets to **A)** collagen and **B)** thrombin. **C)** Effect of SMIFH2 on percentage aggregation at 5 min in response to collagen and thrombin. N=3 for each bar. **D)** Effect of SMIFH2 on ATP secretion in response to collagen and thrombin. N=3 for each bar. **E)** Example flow cytometry traces for platelets treated with 0 or 5 μM SMIFH2 and either stimulated with PBS or thrombin. **F)** Quantification of flow cytometry data showing the effect of SMIFH2 on α-granule secretion and integrin activation.

Supplementary figure 5: A) Effect of SMIFH2 on the final surface area coverage of platelet thrombi on collagen. **B)** Effect of SMIFH2 on the rate of platelet thrombi formation during the flow experiment. **C)** Example images showing the effect of SMIFH2 on spreading of human platelets on fibrinogen from either Tyrode's (washed platelets – upper panel) or platelet rich plasma (lower panel). Scale bars = 10 μm. **D)** Effect of SMIFH2 on the final surface area coverage of pre-formed platelet thrombi after 20 mins of washing in buffer containing SMIFH2. **E)** Comparison of the effect of SMIFH2 on the change in size of individual thrombi after 20 min of washing in buffer containing SMIFH2.

Supplementary figure 6: E **A)** Full western blots for data in Figure 6. **B)** Individual quantitation of acetylated tubulin and tyrosinated tubulin used to determine Ac-tub:Tyr-tub ratios in Figure 6. Data is mean ± SEM.

Supplementary figure 1

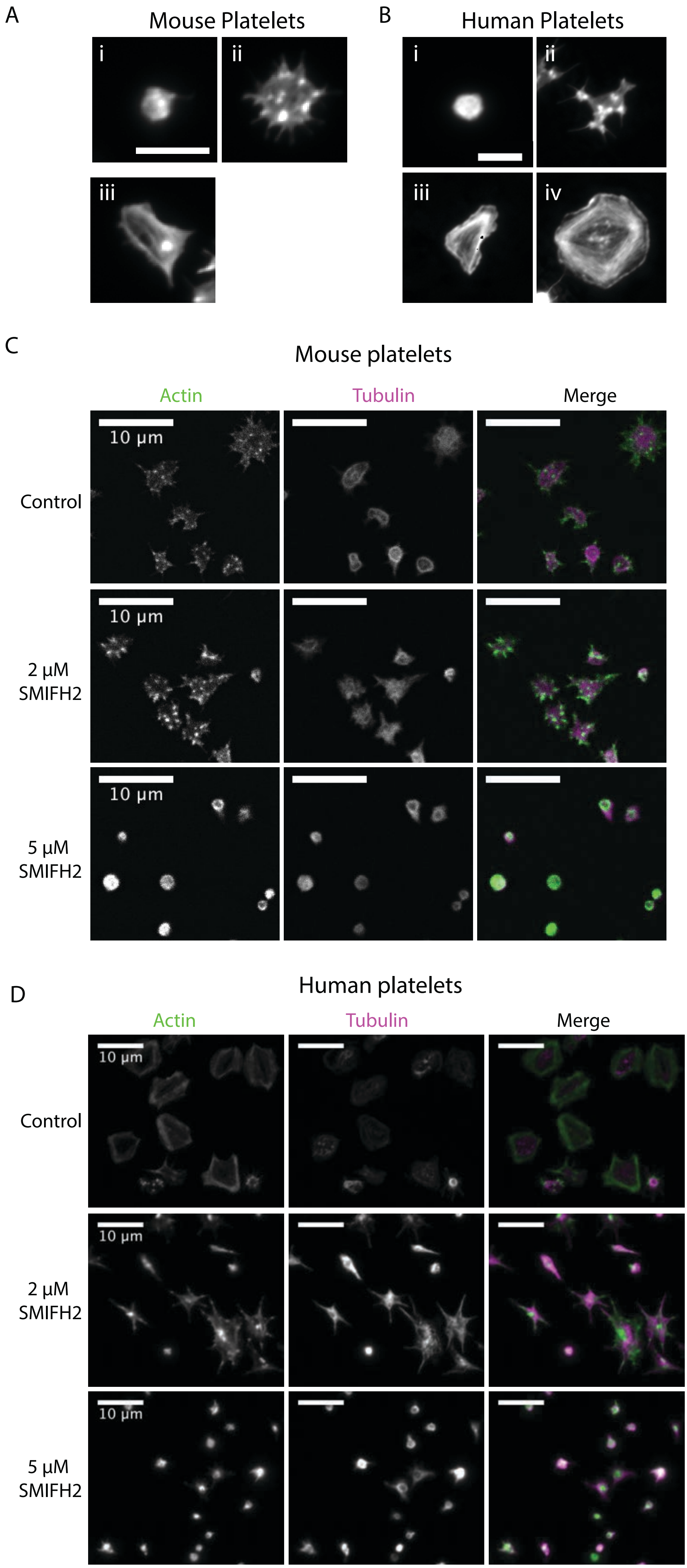

Supplementary figure 2

A

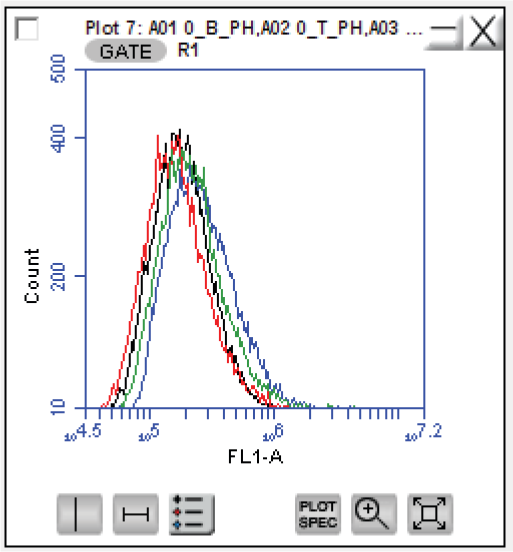

B

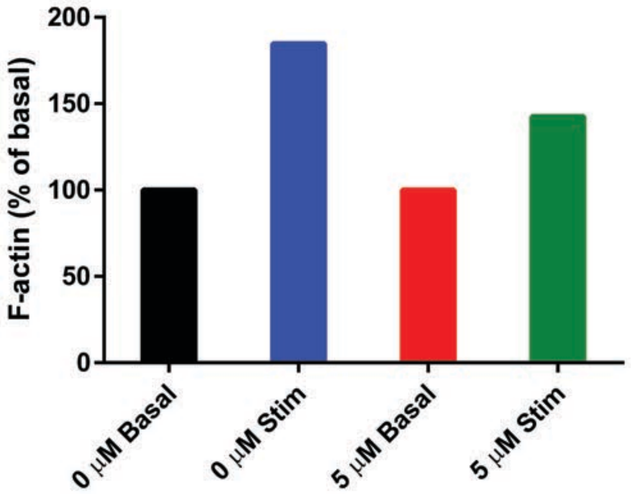

Supplementary figure 3

A

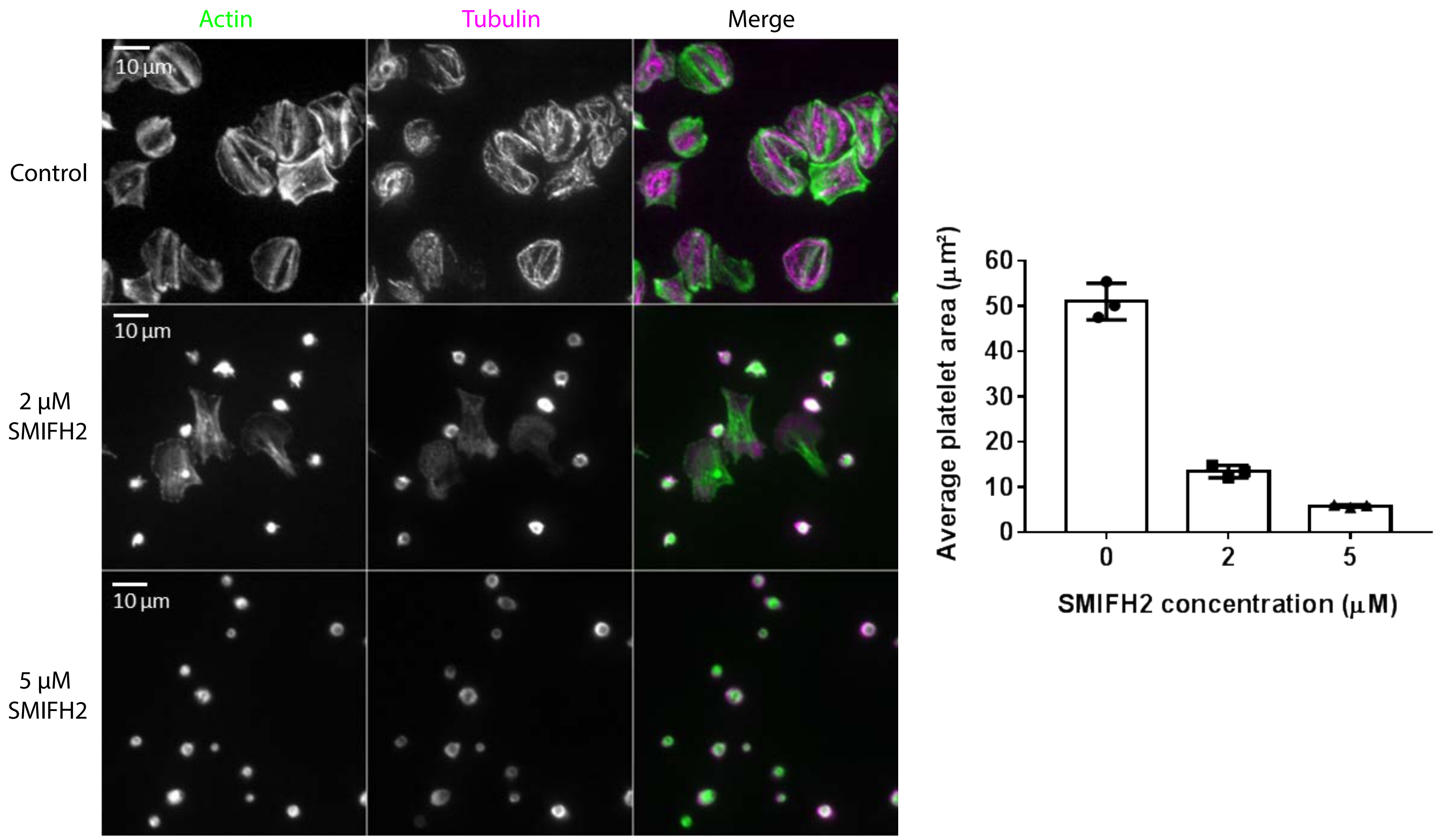

B

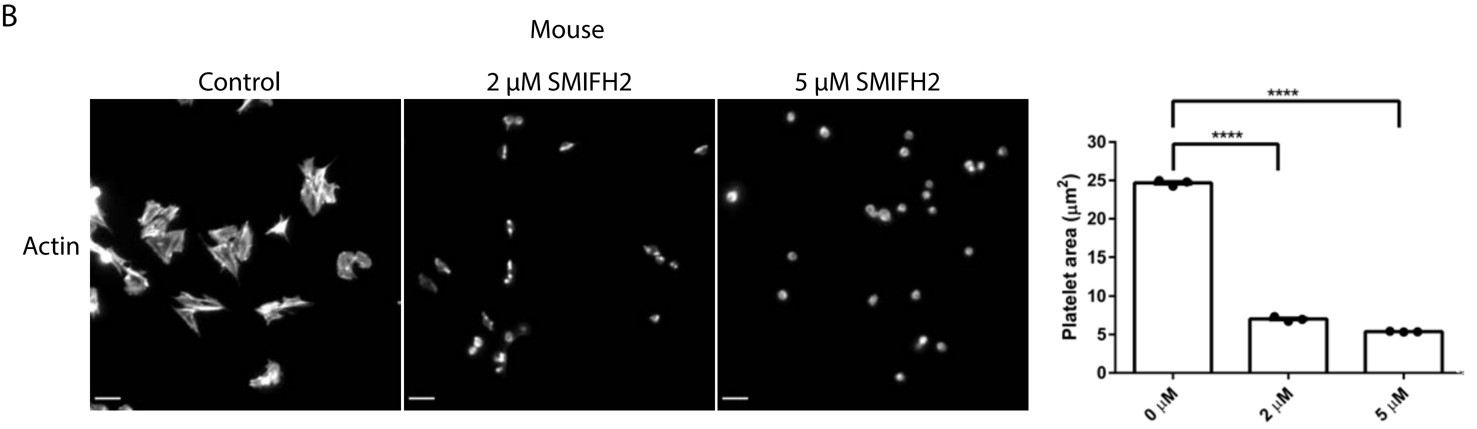

C

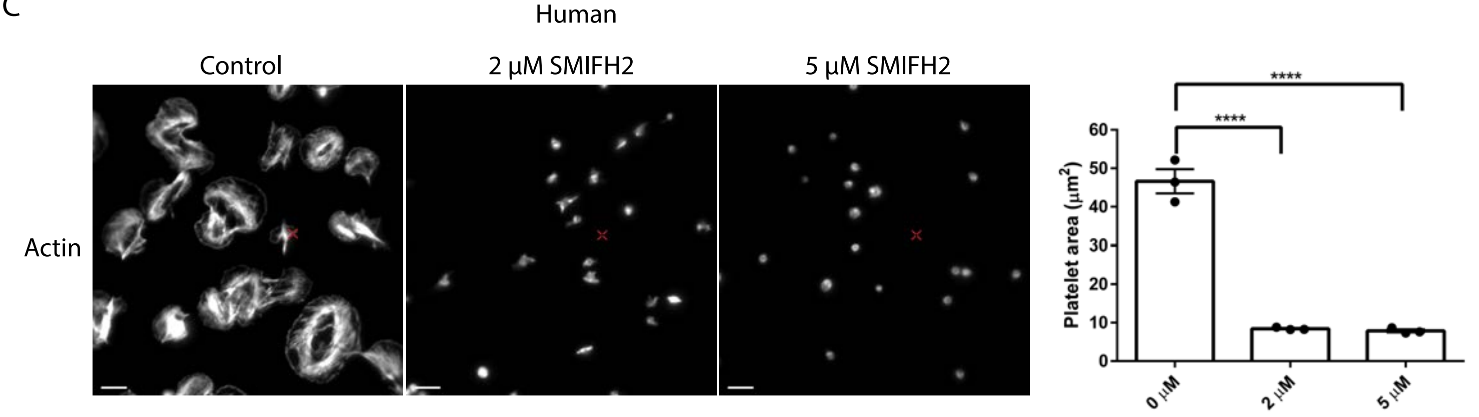

Supplementary figure 4

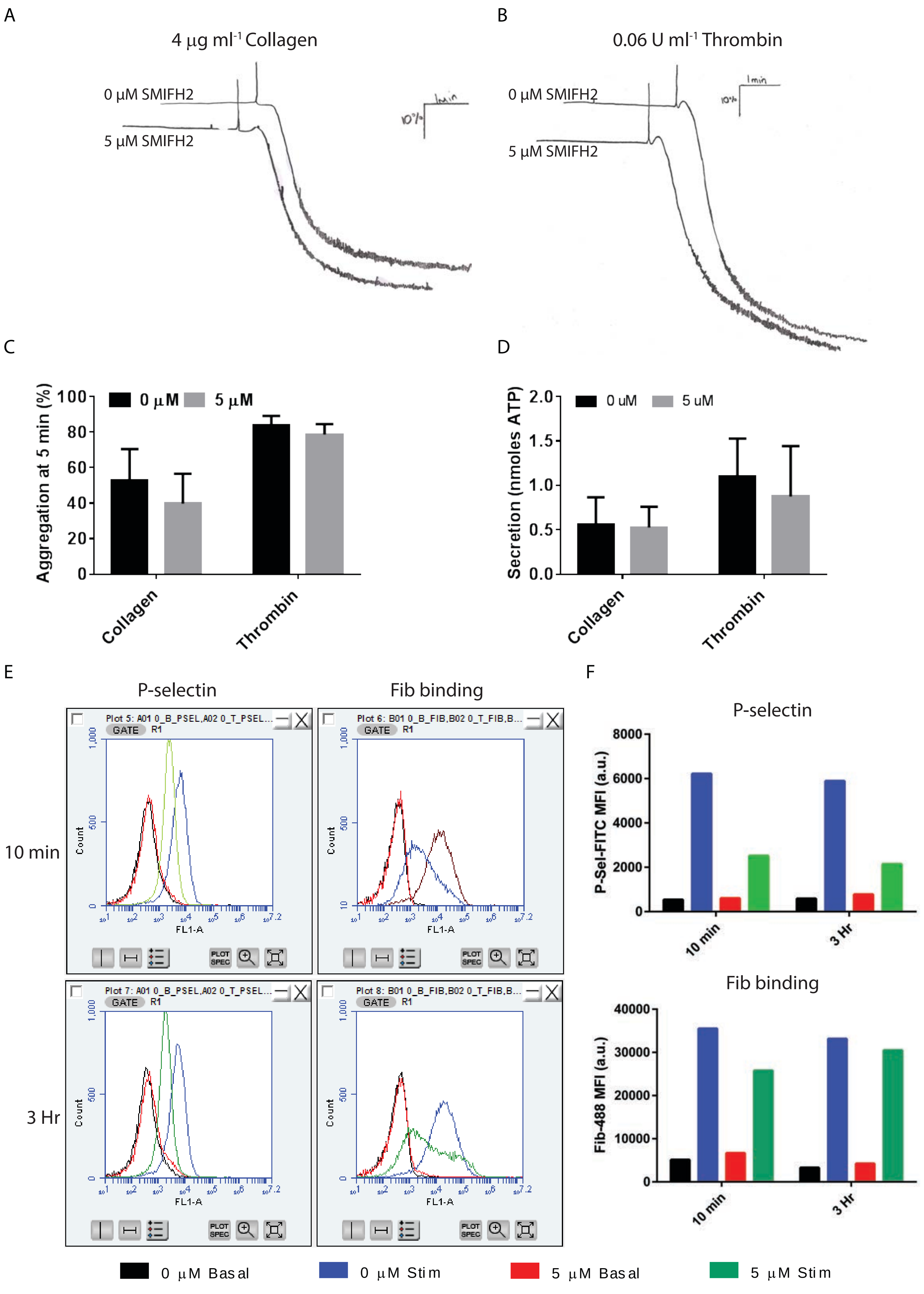

Supplementary figure 5

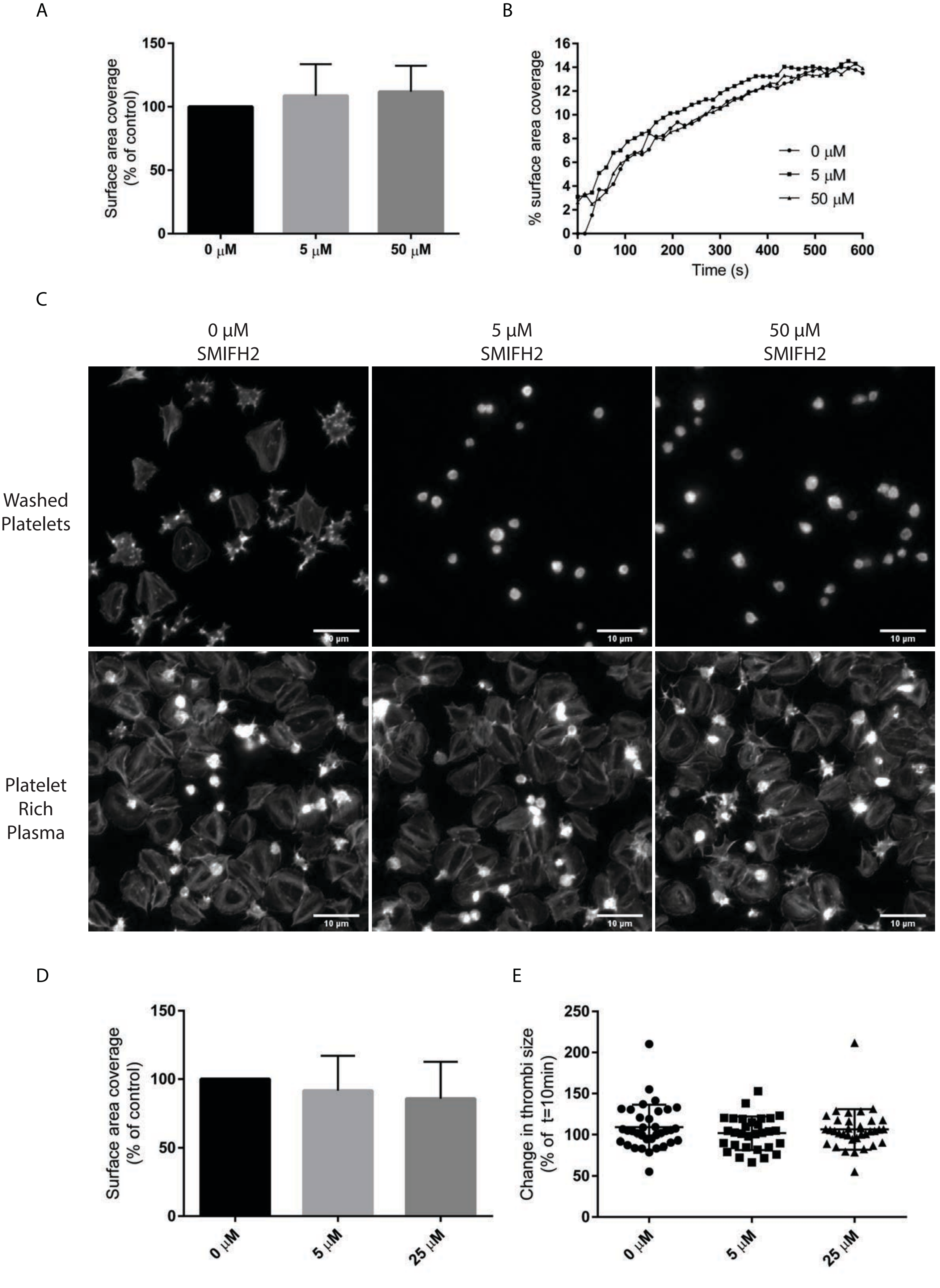

Supplementary figure 6

A

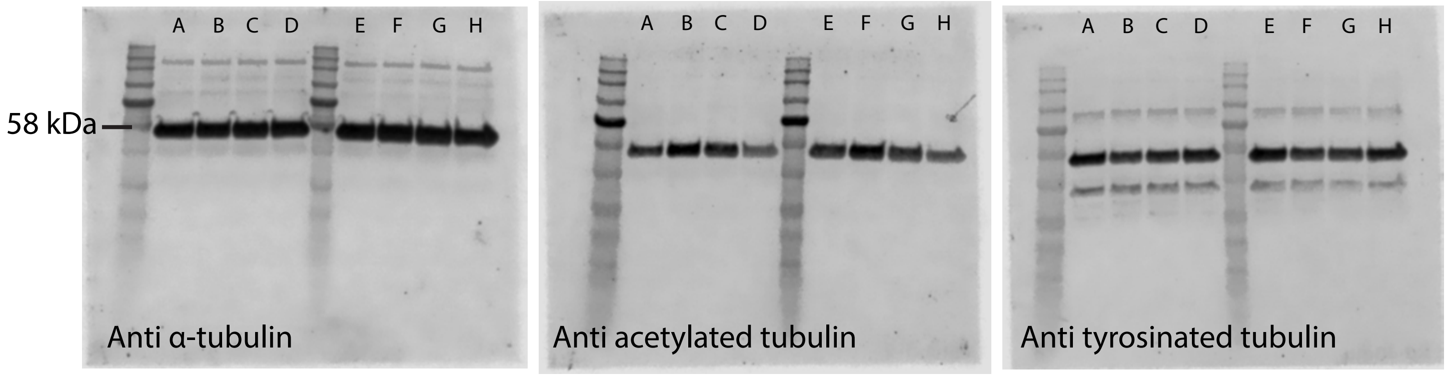

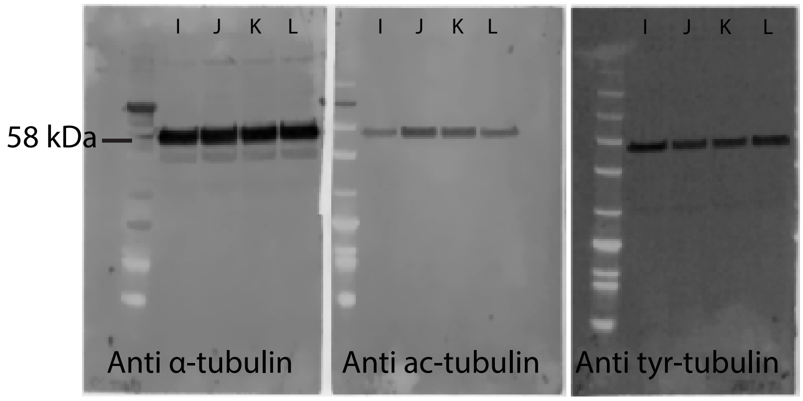

A - Postive control 1
B - 0 μM repeat 1
C - 2 μM repeat 1
D - 5 μM repeat 1
E - Positive control 2
F - 0 μM repeat 2
G - 2 μM repeat 2
H - 5 μM repeat 2

I - Postive control 3
J - 0 μM repeat 3
K - 2 μM repeat 3
L - 5 μM repeat 3

B

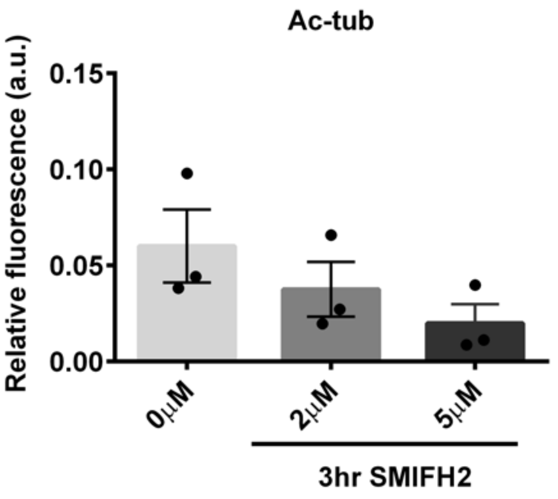

C

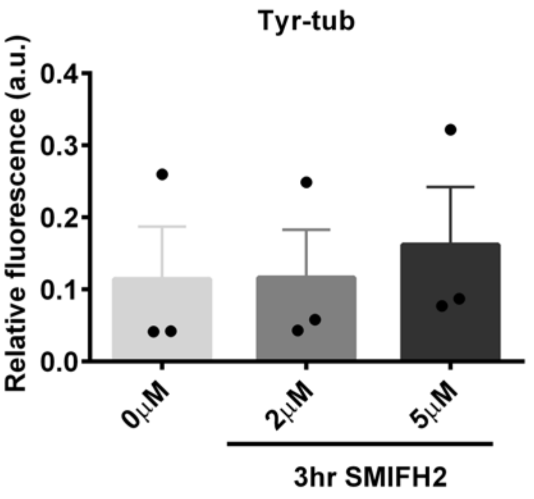

Supplement: Supplementary file 1 [file JTH-18-955-s001.pdf]
